# Rapidly growing juvenile granulosa cell tumor of the ovary arising in adult: a case report and review of the literature

**DOI:** 10.1186/s13048-018-0474-0

**Published:** 2018-12-14

**Authors:** Yuki Inada, Go Nakai, Kazuhiro Yamamoto, Takashi Yamada, Yoshinobu Hirose, Yoshito Terai, Masahide Ohmichi, Yoshifumi Narumi

**Affiliations:** 1 Department of Radiology, Osaka Medical College, 2-7 Daigaku-machi, Takatsuki City, Osaka, 569-8686 Japan; 2 Department of Pathology, Osaka Medical College, 2-7 Daigaku-machi, Takatsuki City, Osaka, 569-8686 Japan; 3 Department of Obstetrics and Gynecology, Osaka Medical College, 2-7 Daigaku-machi, Takatsuki City, Osaka, 569-8686 Japan

**Keywords:** Granulosa cell tumor, Juvenile granulosa cell tumor, Ovary, Adult, MRI, Growth rate

## Abstract

**Background:**

Ovarian granulosa cell tumors (GCTs) are divided into adult GCT (AGCT) and juvenile GCT (JGCT). The AGCT is more common type, conversely, less than 5% of tumors are the JGCT and occur in mainly premenarchal girls and in women younger than 30 years. Although JGCT have different histologic features compared to AGCT, the two types have similar imaging features because they have similar gross appearance. Therefore, it is difficult to distinguish two types by radiologic findings. In addition, it has not been described about the growth rate of JGCTs in past literatures. The aims of this report were to describe a case of rapidly growing JGCT arising in adult with difficulty in diagnosing and to review the literatures.

**Case presentation:**

A 38-year-old woman, presented with abdominal distension and frequent urination, was found to have a pelvic mass measuring approximately 12 cm on ultrasonography. On magnetic resonance imaging (MRI), right ovarian multiloculated cystic mass accompanied with hemorrhagic foci was demonstrated. Although the presumptive diagnosis of GCT was made based on MR findings, the intraoperative differential diagnoses included GCT, yolk sac tumor or malignant mucinous tumor due to cytologic atypia and lack of the typical findings for AGCT such as nuclear grooves and Call-Exner bodies. As a result, abdominal simple total hysterectomy, bilateral oophoro-salpingectomy, partial omentectomy and appendectomy were performed. Moreover, she had a history of laparoscopic uterine myomectomy about one year before, and during that surgery bilateral ovaries were found to be macrospically normal. Therefore, it was suspected the tumor became enlarged within the short period of time.

**Conclusions:**

Even though it is difficult to distinguish two types of GCT by imaging findings, in some cases without typical findings for AGCT pathologically, MRI could provide useful information in accurately diagnosing JGCT. Moreover, in this case, the tumor growth rate seemed to be rapid regardless of its borderline malignant potential. It may be related with nuclear atypia and high mitotic rate of the tumor.

## Background

Granulosa cell tumors (GCTs) are rare sex cord-stromal tumors, encompassing 1–5% of all ovarian tumors [1]. These tumors are divided into adult GCT (AGCT) and juvenile GCT (JGCT) [2]. The AGCT is the more common type, accounting for nearly 95% of all GCTs. They are usually present in their 40’s or above [3]. Conversely, less than 5% of tumors are the JGCT and occur in mainly prepubescent girls and in women younger than 30 years [2]. Histologically, Call- Exner bodies which are gland-like structures resembling ovarian follicles, and grooved, pale, round nuclei called “coffee-bean” nuclei displaying a low mitotic rate are classic features of AGCT. On the other hand, there are few typical findings for AGCT, in addition the immature nuclei show atypia with increased mitotic activity in JGCT [2]. Although two types of GCT have different clinical and histologic features, they have similar imaging features because they have similar gross appearance [1, 4]. So there may be discrepancy between diagnostic imaging and pathological diagnosis in adult patients of JGCT. The growth rate of JGCT is considered more slowly because it is borderline malignant tumor. Here, we report a case of rapidly growing JGCT in adult patient.

## Case presentation

**Fig. 1:**
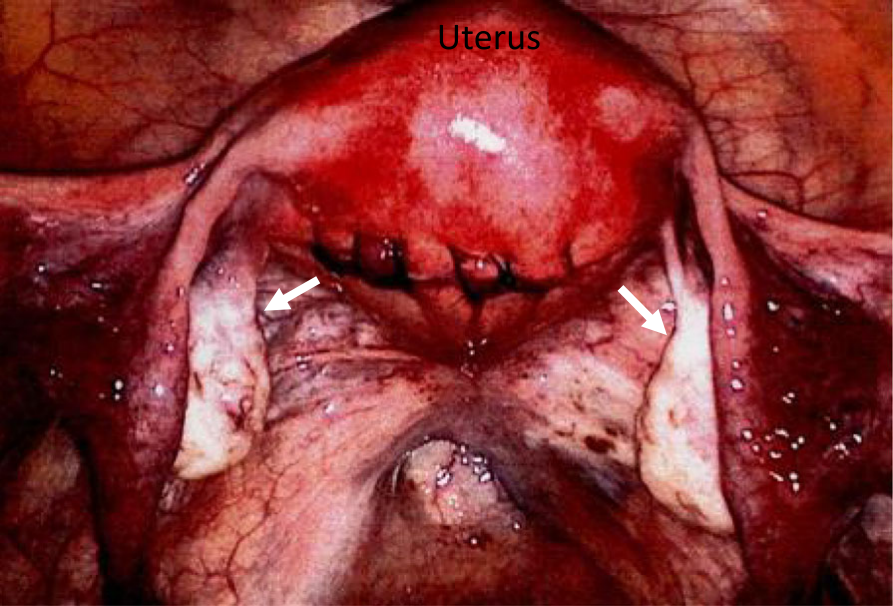
Findings at laparoscopic myomectomy performed about one year before the current episode. Both ovaries appear to be normal (arrows)

A 38-year-old Japanese woman, gravida 0, presented with abdominal distension and frequent urination, was found to have a pelvic mass on radiologic examinations. She had a history of laparoscopic uterine myomectomy about a year before the onset, where the bilateral ovaries were macroscopically normal (Fig. 1). She had no menstrual irregularities or dysfunctional uterine bleeding. Serum estradiol (E2) level was elevated to 214.5 pg/ml (normal 70–160 pg/ml), while testosterone was within the normal range. Luteinizing hormone (LH) and follicle stimulating hormone (FSH) were 2.0 mIU/ml (normal 1-14mIU/ml) and less than 0.1 mIU/ml (normal 1.5-8mIU/ml) respectively, indicating E2-mediated negative feedback. CA 125 level was slightly elevated to 39.2 U/ml (normal < 35.0 U/ml). CEA, CA 19–9 and SCC antigen were within the normal range.

**Fig. 2:**
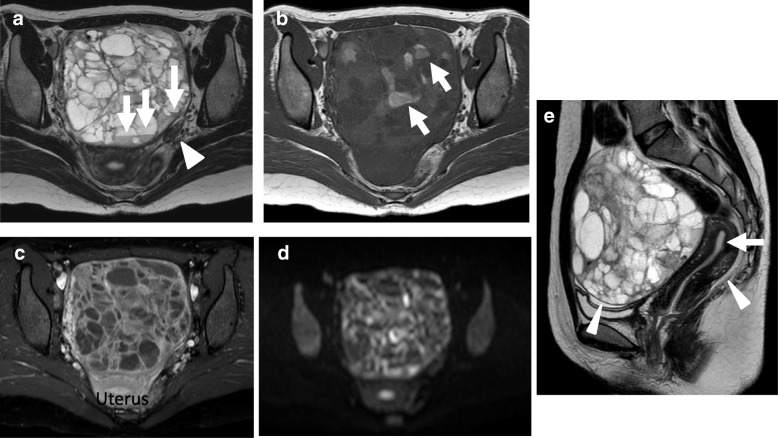
On MR images, a large, multiloculated right ovarian cystic mass, approximately 10 × 10 × 12 cm in size, was located at front of the uterus. **a** Axial T2-weighted image shows the multiloculated cystic lesion accompanied with thick septations. Fluid-fluid levels are demonstrated in several cystic components (arrows). The atrophic left ovary with few follicles is also detected (arrowhead). **b **Axial T1 weighted image shows intracystic high signal intensities suggesting intracystic hemorrhage (arrows). **c **On the contrast-enhanced axial fat-suppressed T1-weighted image, the septations demonstrated strong contrast enhancement similar to that of uterine myometrium. **d** Axial diffusion-weighted image demonstrates the septations having high signal intesnsities. **e** On sagittal T2 weighted image the uterus is normal size without endometrial thickening (arrow). There is a small amount of ascites which is limited to the pouch of Douglas and vesicouterine pouch (arrowheads)

The MR showed a large, multiloculated cystic mass with numerous septations in the pelvis measuring approximately 10 × 10 × 12 cm. On T2-weighted images, fluid-fluid levels were demonstrated in several cystic components (Fig. 2a). T1-weighted images demonstrated intracystic high signal intensities suggesting intracystic hemorrhage (Fig. 2b). Contrast-enhanced fat-suppressed T1-weighted images showed strong enhancement of the septations similar to uterine myometrium (Fig. 2c). The mass was suspected to originate from the right ovary because the right ovary was not identified. The left ovary was atrophic for her age (Fig. 2a). On diffusion-weighted imaging, the septations showed high signal intensity (Fig. 2d). The uterus was of normal size without endometrial thickening. There was a small amount of ascites which was limited to the pouch of Douglas and vesicouterine pouch (Fig. 2e). Any calcifications were not detected on CT images. GCT was suspected from these findings.

Abdominal right ovarian tumor resection was performed. During the operation, the frozen section of the right ovarian tumor showed that malignancy could not be excluded due to its nuclear atypia. The differential diagnoses of the tumor included yolk sac tumor, malignant mucinous tumor and AGCT despite of the lack of any typical findings such as coffee-bean nuclei and Call-Exner bodies. Based on this report, abdominal simple total hysterectomy, bilateral oophoro-salpingectomy, partial omentectomy and appendectomy were performed.

**Fig. 3:**
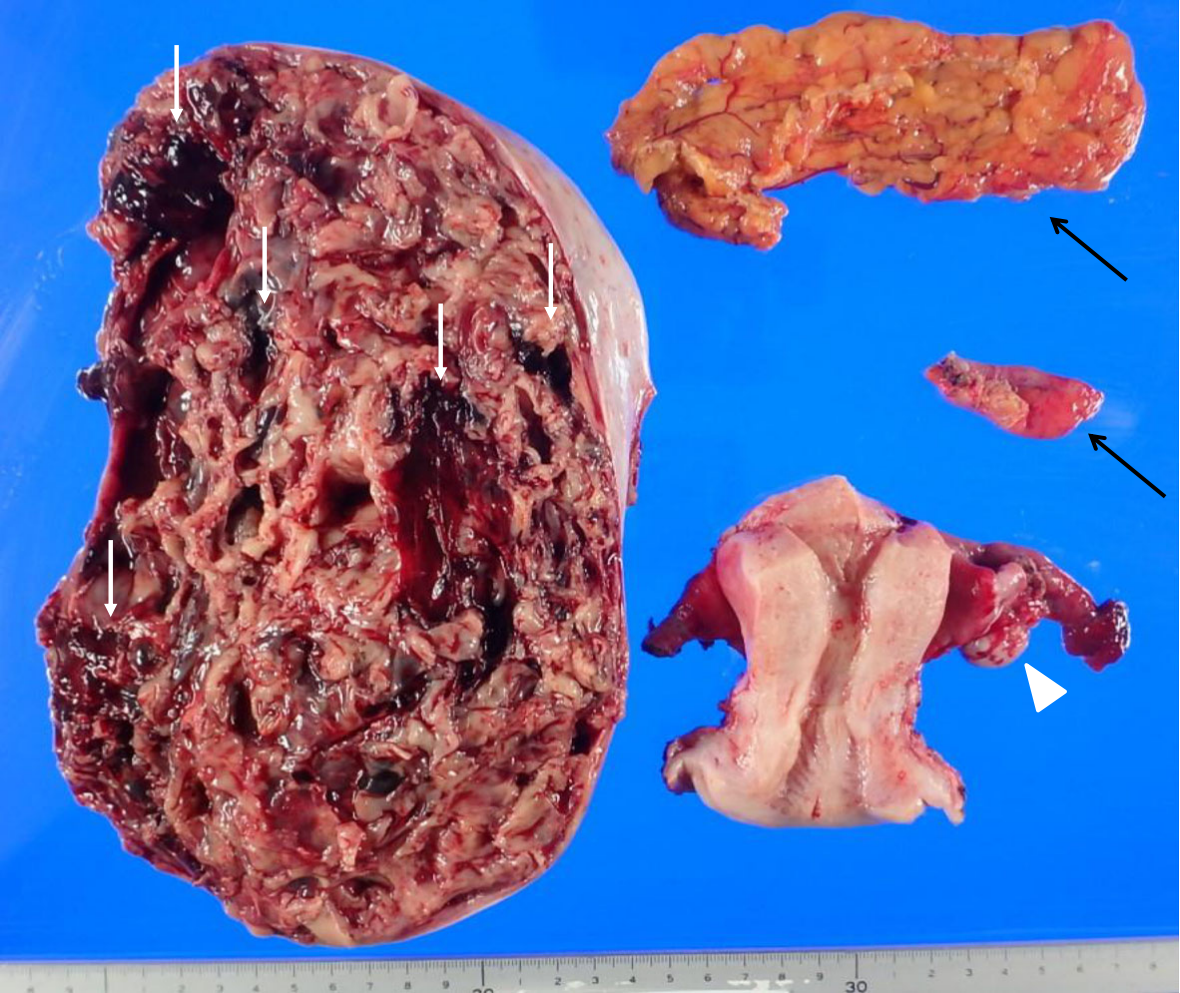
Gross appearance of the resected specimens. The cut surface of the right ovarian tumor measuring 13 cm in diameter shows multiloculated cystic tumor accompanied by intracystic hemorrhagic foci (white arrows). The left ovary has the maximum diameter of 1.7 cm suggesting atrophy for her age macroscopically (arrowhead). Partial omentectomy and appendectomy were performed (black arrows)

**Fig. 4:**
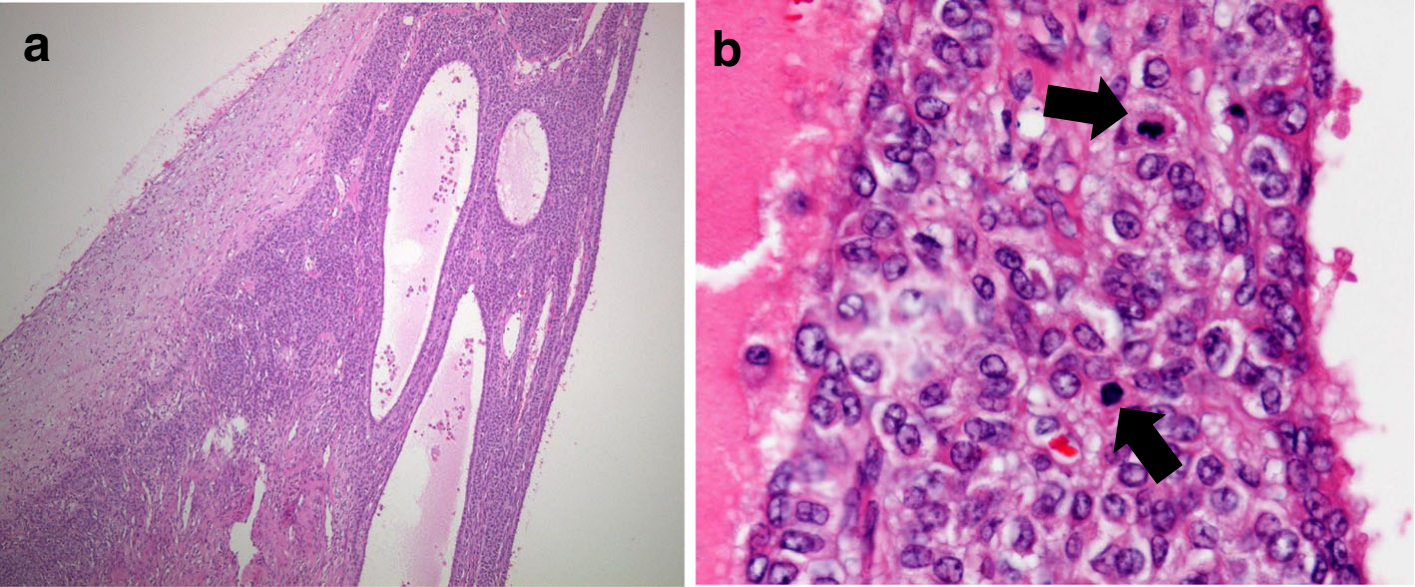
Photomicrograph of the right ovarian tumor. **a** It demonstrates round cells surrounded the macrofolliculars with eosinophilic material and hemorrhage. **b** The mitotic activity is observed (arrows) and focally brisk, with an average of 10 mitoses per high-power field in the areas

The gross appearance of the cut surface of the right ovarian tumor, measuring 13 cm in diameter, showed multiloculated cystic tumor accompanied by intracystic hemorrhagic foci. The left ovary had a maximum diameter of 1.7 cm, suggesting atrophy for her age (Fig. 3). Microscopic examination of the right ovary showed round cells that surrounded the macrofolliculars with eosinophilic material and hemorrhage (Fig. 4a).The tumor cells had scant cytoplasm, round-to-oval vesicular nuclei with small eosinophilic nucleoli, and irregular nuclear contours. The typical findings for AGCT such as longitudinal nuclear grooves (coffee-bean nuclei) and Call-Exner bodies were not identified. The mitotic activity was focally brisk, with an average of 10 mitoses per 10 high-power fields in these areas (Fig. 4b). Immunohistochemically, tumor cells were positive for vimentin, calretinin, CD99, a-inhibin and MIB-1 labeling index was about 30%. The above findings supported the diagnosis of JGCT. Accordingly, the definitive diagnosis of JGCT, FIGO Stage IA led to no additional treatment.

## Discussion

GCTs are rare sex cord-stromal tumors, encompassing 1–5% of all ovarian tumors [1]. These tumors are divided into AGCT and JGCT, reflecting not only the typical age of presentation, the differentiating histologic characteristics but also the differing natural history [2]. The AGCT is the more common type, accounting for nearly 95% of all GCTs. They are usually present in women in their 40’s or above [3]. Conversely, less than 5% of tumors are the JGCT and occur in mainly prepubescent girls and in women younger than 30 years, with a mean age of 13 years. In one series, 97% of cases were present before 30 years of age [2].

Histologically, Call-Exner bodies which are gland-like structures resembling ovarian follicles, and “coffee-bean” nuclei, which are grooved, pale and round nuclei displaying a low mitotic rate are classic features of AGCTs, while gland-like structures resembling ovarian follicles in JGCTs are irregular in size and shape. In addition, the immature nuclei show atypia with increased mitotic activity in JGCTs [2]. A positive immunohistochemical stain for a-inhibin, an ovarian glycoprotein, is a key diagnostic feature for GCTs.

Grossly, AGCTs can be cystic (30.3%), solid (27.8%) or solid and cystic (41.7%) [5], while JGCTs show similar gross feature, that are cystic (14%), solid (37%) and solid and cystic (45%) [2]. Although JGCTs have different clinical and histologic features compared to AGCTs, the two types have similar imaging features because they have similar gross appearance [1, 4]. GCTs have some distinctive MR imaging features: a sponge-like appearance with solid areas of intermediate signal intensity and numerous cystic spaces on T2-weighted MR images, and hemorrhagic foci of high signal intensity on T1-weighted MR images. It also can manifest as a solid mass with variable cystic areas or as a predominantly cystic mass with solid portions. Uterine enlargement or endometrial thickening may be seen as a result of estrogenic effect [1, 6, 7]. Although a few literatures described MR findings of AGCTs, a JGCT typically appears at imaging as a large, unilateral, multicystic mass with a solid portion and sometimes with irregular septa [5, 8, 9].

In this case, the tumor showed multiloculated cystic mass with intracystic hemorrhage on the MRI. On diffusion-weighted images (DWIs), the septations of tumor demonstrated high signal intensities (SIs). The feature was consistent with the previous result reporting that DWIs showed high SIs in the solid component of the GCT [10]. Although these findings were considerable for both AGCT and JGCT, there were no typical features of AGCT in a frozen section of the tumor, such as Call-Exner bodies and “coffee-bean” nuclei. In addition, taking the patient’s age into consideration, it was more difficult to reach a correct diagnosis during the operation. A similar case of JGCT has been described in a 43-year-old woman in a previous study [11]. As a result, the discrepancy between the diagnosis based on MR findings and intraoperative pathologic results may lead to difficulty in choosing appropriate treatment in adult patients with JGCTs.

The most common presenting symptoms of both AGCT and JGCT are abdominal pain and increasing abdominal girth [12]. The produced estrogen induces precocious puberty in 10% of premenarchal females as JGCT can be hormonally active [13]. Dysfunctional uterine bleeding and menstrual irregularities are frequently seen in women of reproductive age with hormonally active GCTs [12]. However, this case did not show menstrual irregularities despite of the tumor secretion of E2 but showed the atrophic contralateral ovary on gross appearance as well as on the MRI due to suppression of the serum LH and FSH levels. In women of reproductive age, it is not easy to assume if the ovarian tumor produces E2 or not on MRI, although the enlarged uterus, thickened endometrium and the typical symptom such as postmenopausal bleeding can be referred as signs of estrogen-producing ovarian tumor in postmenopausal women. However, the atrophic change in the normal side ovary observed in this case can be one of the clues in terms of assuming the hormonal activity of the ovarian tumor.

Another striking feature in this case was the growth rate of the tumor regardless of its borderline malignant potential. Any macroscopic abnormalities in the bilateral ovaries in the previous surgery about one year before were not observed. Therefore, it was suspected the tumor became enlarged within the short period of time. It has not been described about the growth rate of JGCTs in past literatures. It may be related with nuclear atypia and high mitotic rate of the tumor.

## Conclusions

In conclusion, even though JGCT in adult patients could be correctly diagnosed as GCT preoperatively, intraoperative pathological diagnosis may be difficult. MRI could provide useful information in accurately diagnosing JGCT. Moreover, it was suspected the tumor became enlarged within the short period of time in this case. It may be related with nuclear atypia and high mitotic rate of the tumor.

## Abbreviations

AGCT: Adult granulosa cell tumor
CA 125: Carbohydrate antigen-125
CA 19–9: Carbohydrate antigen 19–9
CD99: Cluster of differentiation 99
CEA: Carcinoembryonic antigen
DWI: Diffusion-weighted image.
E2: Estradiol
FIGO: International Federation of Gynecology and Obstetrics
FSH: Follicle stimulating hormone
GCT: Granulosa cell tumor
JGCT: Juvenile granulosa cell tumor
LH: Luteinizing hormone
MRI: Magnetic resonance imaging
SCC: Squamous cell carcinoma
SI: Signal intensity
